# Supplementary material for: Thioredoxin Reductase Activity Predicts Gold Nanoparticle Radiosensitization Effect
Source: Nanomaterials (Basel). 2019 Feb 19;9(2):295. doi: 10.3390/nano9020295 (PMC6409576; doi:10.3390/nano9020295)
Supplement: Supplementary file 1 [file nanomaterials-09-00295-s001.pdf]

Thioredoxin Reductase Activity Predicts Gold Nanoparticle Radiosensitization Effect

Sébastien Penninckx [1], Anne-Catherine Heuskin [1], Carine Michiels [2,*] and Stéphane Lucas [1]

[1] Research center for the Physics of Matter and Radiation (PMR-LARN), Namur Research Institute For Life Sciences (NARILIS), University of Namur, Rue de Bruxelles 61, B-5000 Namur, Belgium; sebastien.penninckx@unamur.be (S.P.); anne-catherine.heuskin@unamur.be (A.-C.H.); stephane.lucas@unamur.be (S.L.)

[2] Unité de Recherche en Biologie Cellulaire (URBC), Namur Research Institute For Life Sciences (NARILIS), University of Namur, Rue de Bruxelles 61, B-5000 Namur, Belgium

* Correspondence: carine.michiels@unamur.be; Tel.: +32-81-724131; Fax +32-81-724135

Supplementary information

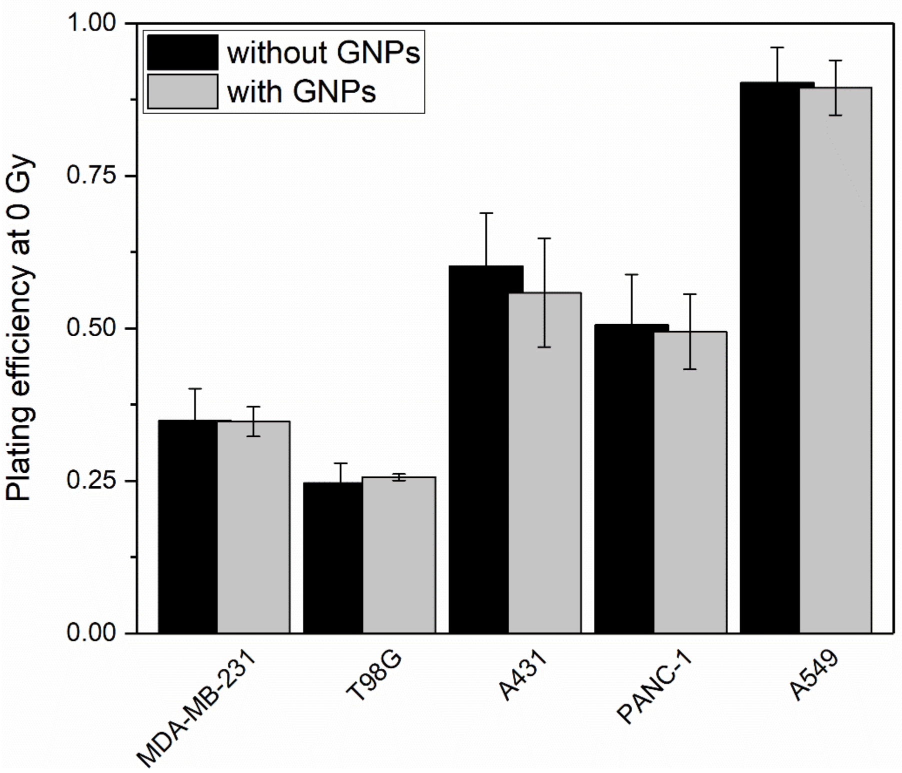

Figure S1. Toxicity of GNPs across the cell lines. All cells were incubated 24 h with 50 µg/mL GNPs prior the clonogenic assay. Results were reported as mean plating efficiency ± SEM. Results were statistically analyzed using a one-way ANOVA evidencing no significant difference (Tukey test).

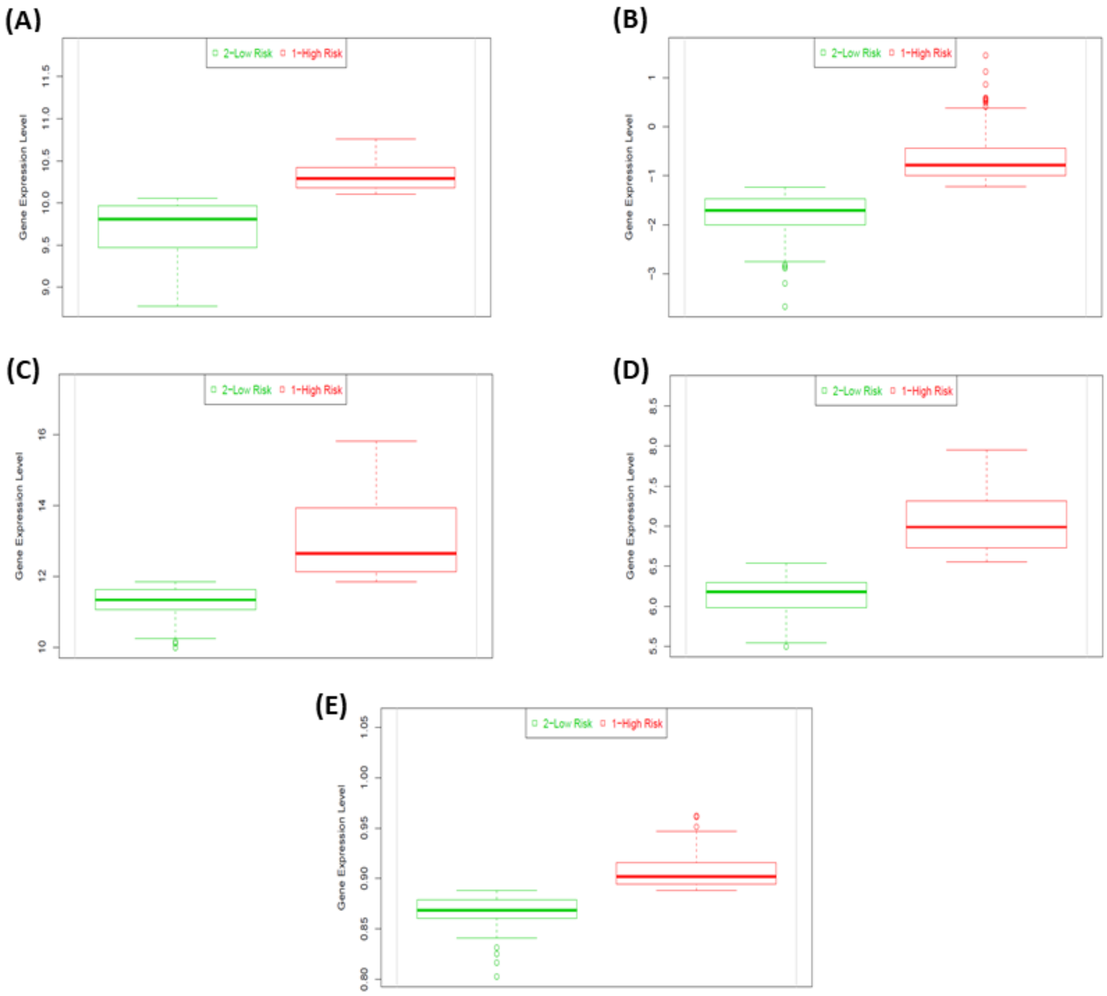

Figure S2. Box plot of TXRND1 mRNA expression across the two expression groups for (A) brain (GSE42669), (B) breast (TCGA-BRCA), (C) head & neck (TCGA-HNSC), (D) lung (GSE 30219) and (E) pancreas (PACA-AU) cancer datasets. P-values were calculated using t-test.
